# Comment on ‘Capecitabine and bevacizumab as first-line treatment in elderly patients with metastatic colorectal cancer’

**DOI:** 10.1038/sj.bjc.6606037

**Published:** 2010-12-14

**Authors:** M J Molina-Garrido, C Guillén-Ponce

**Affiliations:** 1 Medical Oncology Section in the Hospital Virgen de la Luz in Cuenca, Hermandad Donantes de Sangre Street, Cuenca, CP: 16002, Spain; 2 Medical Oncology Service in the Hospital Ramón y Cajal in Madrid, Madrid, Spain

**Sir,**

We read with great interest a recently published article in your journal titled ‘Capecitabine and bevacizumab as first-line treatment in elderly patients with metastatic colorectal cancer’ (Feliu, 2010).

Initially, I would like to commend the authors of that article, especially for their interest in finding effective treatment regimens for the elderly, which are also associated with acceptable tolerance levels.

However, I would like to highlight certain data.

People aged 65 years and older have a cancer incidence 11 times greater than that of younger individuals, and the risk of mortality from malignancy is 16 times higher (http://seer.cancer.gov/csr/1975_2000/). Demographic shifts are producing a very rapid growth in at-risk populations, so that by 2030, 20% of the population will be over 65 years. Unfortunately, oncologists are not sufficiently prepared for this demographic shift, as their training focuses on selecting the best therapeutic approaches for young and physically healthy patients (Mandelblatt *et al*, 2000; Hurria *et al*, 2003). There is, however, significant heterogeneity among elderly patients, even among those with the same chronological ages. Such heterogeneity is associated with different tolerance levels towards cancer treatments.

Oncologists need an assessment tool that will provide information about the ‘functional age’ of older individuals, rather than the ‘chronological age’. An assessment tool named the ‘comprehensive geriatric assessment’ (CGA) may help to identify elderly patients who are most vulnerable to complications from cancer treatments. This interdisciplinary assessment provides information about the patient's functional status, comorbidity, nutritional status, psychological status, social support, cognitive status and other medications (Extermann *et al*, 1998; Repetto *et al*, 2002; Extermann and Hurria, 2007).

Several cross-sectional studies have demonstrated an association between the CGA and factors such as toxicity, morbidity and mortality during cancer treatment in older patients (Extermann *et al*, 2002; Audisio *et al*, 2005; Freyer *et al*, 2005; Maione *et al*, 2005; Ramesh *et al*, 2005).

In the field of geriatric oncology, the CGA can distinguish three broad groups of elderly patients: (1) ‘fit’ patients, who can be treated with chemotherapy in the same way as younger patients; (2) ‘prefrail’ patients, for whom chemotherapy should be administered with special schemes, reduced doses and haematological support factors; and (3) ‘frail’ patients, for whom the best therapeutic option involves supportive care and nonspecific palliative treatment (Balducci, 2007). However, the authors of the article merely utilised three parameters to decide whether it was possible to administer chemotherapy to the elderly: functional status, as measured by the Lawton–Brody Scale and the Barthel Scale; comorbidity, as measured by the Charlson Index; and the researchers' own subjective opinions. Recently, it has been reported that low scores on the ‘Mini Nutritional Assessment’ (MNA) questionnaire, which is used to assess nutritional status, and on the ‘Mini Mental State Examination’ (MMSE), which is used to determine cognitive status, are associated with an increased likelihood of elderly patients being unable to complete chemotherapy. In addition, a low score on the MNA is associated with an increased risk of mortality if chemotherapy is administered to the elderly (Aaldriks *et al*, 2010). Such findings indicate that there are sufficient functional status assessment options for elderly patients with cancer.

Based on these data, we strongly advocate that the CGA be used to evaluate elderly patients before the administration of any cancer treatment. Although only a few authors have used specific models of the CGA (Balducci, 2001; Ingram *et al*, 2002; Repetto *et al*, 2002; Hurria *et al*, 2005; Overcash *et al*, 2006; Molina-Garrido and Guillén-Ponce, 2010), any of these models could have been applied to this study.

We also believe that the subjective data, though important in subject areas with limited previous research, should be relegated to the background, especially because there is an objective way to evaluate elderly cancer patients: the CGA.
